# Influence of aromatic essential oil nursing on mental state of patients undergoing laparoscopic cholecystectomy: A protocol for systematic review and meta-analysis

**DOI:** 10.1097/MD.0000000000029122

**Published:** 2022-04-01

**Authors:** Xin Liu, Yingni Luan, Lin Zhang, Yanli Li

**Affiliations:** a Department of Operating Room, Tongji Hospital of Tongji Medical College of Huazhong University of Science and Technology, Wuhan, China; b Department of Traditional Chinese Medicine Physiotherapy, Shandong University Hospital, Shandong, China; c Department of Surgery, Liaocheng Third People's Hospital, Shandong, China; d College of Health, Binzhou Polytechnic, Shandong, China.

**Keywords:** anxiety, aromatic essential oil nursing, laparoscopic cholecystectomy, meta-analysis, protocol

## Abstract

**Background:**

There are no evidence-based data to confirm the efficacy of aromatic essential oil nursing in patients after laparoscopic cholecystectomy (LC). Therefore, in order to provide new evidence-based medical evidence for clinical treatment, we undertook this protocol for systematic review and meta-analysis to assess the effectiveness and safety of aromatic essential oil nursing on mental state of patients undergoing LC.

**Methods:**

The retrieval strategy will be developed by the evidence-based medicine librarians for the US Library of Medicine database, the Cochrane Database of Systematic Reviews, and the Excerpta Medica database. The databases will be searched between June 2022 and July 2022. Studies will be included according to the following criteria: study population undergoing LC; group with aromatic essential oil nursing compared to a control group; outcome measures including anxiety, salivary cortisol, alpha amylase, and patient satisfaction; randomized controlled trials. All data will be analyzed using R version 3.4.3 to calculate pooled standardized mean differences for outcomes. The Cochrane Collaboration's tool will be used to assess the risk of bias for each included article.

**Results:**

The results of this paper will fill a gap in the literature regarding this project.

**Conclusion:**

We assume that the aromatic essential oil nursing has a positive effect.

**Registration number:**

10.17605/OSF.IO/E5WC9.

## 1 Introduction

Laparoscopic cholecystectomy (LC) was first performed in 1987 and has become a successful surgical procedure for biliary colic, cholecystitis, and cholelithiasis. The increasing prevalence of LC is mainly due to the small surgical scars left by laparoscopic surgery; only a short hospital stay can promote quicker recovery.^[1]^ LC has improved outcomes compared to traditional open surgery and is considered the standard of care for the treatment of cholecystitis. In the United States, there are approximately 750,000 LCs per year, and the trend is expected to increase.^[2,3]^

Preoperative anxiety is a common phenomenon in patients undergoing preoperative evaluation. It refers to the process of gradually strengthening from the start of a specific operation to the beginning of the operation. Overall, this can be a very disturbing situation for the patient.^[4]^ Anxiety can lead to increased pain, increased analgesic consumption, longer hospital stays, and has a direct impact on healthcare costs. Some complementary and integrative practices, such as nonpharmacological interventions, may improve human physical, psychological, and social dimensions.^[5]^ Common strategies for treating preoperative anxiety include pharmacological and nonpharmacological interventions. Effective nonpharmacological interventions to reduce anxiety include acupuncture, massage therapy, music therapy, guided imagery therapy, and aromatherapy.^[6–8]^

Aromatherapy using essential oils affects patient health and is considered a simple, low-risk, and inexpensive way to reduce patient anxiety.^[9,10]^ However, there are no evidence-based data to confirm the efficacy of aromatic essential oil nursing in patients after LC. Therefore, in order to provide new evidence-based medical evidence for clinical treatment, we undertook this protocol for systematic review and meta-analysis to assess the effectiveness and safety of aromatic essential oil nursing on mental state of patients undergoing LC.

## 2 Materials and methods

### 2.1 Searching strategy

The protocol was prepared according to the Preferred Reporting Items for Systematic Reviews and Meta-Analyses Protocols statement guidelines. This systematic review and meta-analysis has been prospectively registered in the Open Science Framework Registry, No. 10.17605/OSF.IO/E5WC9. The retrieval strategy will be developed by the evidence-based medicine librarians for the US Library of Medicine database, the Cochrane Database of Systematic Reviews, and the Excerpta Medica database. The databases will be searched between June 2022 and July 2022. Ethical approval is not required as the current meta-analysis will be based on previously published studies.

### 2.2 Study selection and selection criteria

Two independent reviewers will screen titles and abstracts and remove duplicate and irrelevant studies. Full-text articles are then assessed for eligibility. Differences will be resolved through discussions among all authors. Studies will be included according to the following criteria: study population undergoing LC; group with aromatic essential oil nursing compared to a control group; outcome measures including anxiety, salivary cortisol, alpha amylase, and patient satisfaction; randomized controlled trials. The following will be excluded: non-randomized controlled trials; detailed data on outcomes were not reported or could not be calculated from the data provided; studies were poorly designed for comparisons; serial publications from the same cohort, with overlapping participants and study designs, also excluded.

### 2.3 Data extraction

Data from selected papers will be extracted independently by two authors and entered into prespecified tables using standardized data extraction forms. The extracted data will be then compared and any disagreements resolved by discussion or arbitration by the senior authors as necessary. Data collection forms will be pilot tested prior to use. We will extract information on study source, number of patients, type of study, patient characteristics, intervention details, comparison group at the time of reporting, previous treatments, follow-up time points, outcomes, and side effects of the intervention. We will also contact the authors of the studies included in our review when we need more information about their analysis or reported results.

### 2.4 Statistical analysis and data synthesis

All data will be analyzed using R version 3.4.3 to calculate pooled standardized mean differences for outcomes. If the study reported the effectiveness of the aromatic essential oil at multiple time points, the last time point is used in the analysis. Because of the different conditions in the same outcome, the corresponding 95% confidence intervals for the pooled effect size are calculated using a random effects model. Heterogeneity is assessed with the *Q* statistic and is considered significant when *I*^2^ > 50% or *P* < .1. Publication bias is assessed using Egger's regression test and graphically represented using Begg's funnel plot when there are ≥10 studies. In Egger's regression test, *P* < .10 is considered significant. Whenever publication bias is discovered, Duvall and Tweedie's pruning and padding approach is applied to supplementary studies that seemed to be missing to enhance symmetry.

### 2.5 Risk of bias and quality assessment

The Cochrane Collaboration's tool will be used to assess the risk of bias for each included article. Each article will be graded based on sequence generation, allocation concealment, blinding of participants and personnel, blinding of outcome assessment, incomplete outcome data, other possible biases, intention-to-treat analysis, selective reporting (undefined, low, or high risk of bias), and baseline characteristics. Risk of bias assessments will be completed by one author and checked by a second author. Differences will be resolved by discussion, and if consensus cannot be reached, a third author will be consulted.

## 3 Discussion

Preoperative anxiety has been reported to be associated with postoperative pain, cognitive impairment, and delayed healing. Stress responses, abdominal surgical interventions, trauma, postoperative pain, and the use of anaesthetics and narcotics are among the factors that contribute to the development of gastrointestinal problems. Aromatherapy using essential oils affects patient health and is considered a simple, low-risk, and inexpensive way to reduce patient anxiety.^[9,10]^ However, there are no evidence-based data to confirm the efficacy of aromatic essential oil nursing in patients after LC. Therefore, in order to provide new evidence-based medical evidence for clinical treatment, we undertook this protocol for systematic review and meta-analysis to assess the effectiveness and safety of aromatic essential oil nursing on mental state of patients undergoing LC.

## Author contributions

**Conceptualization:** Lin Zhang.

**Data curation:** Xin Liu, Yingni Luan.

**Formal analysis:** Xin Liu, Yingni Luan.

**Funding acquisition:** Yanli Li.

**Methodology:** Lin Zhang.

**Project administration:** Yanli Li.

**Resources:** Yanli Li.

**Software:** Xin Liu, Yingni Luan.

**Supervision:** Yanli Li.

**Validation:** Lin Zhang.

**Visualization:** Lin Zhang.

**Writing – original draft:** Xin Liu, Yingni Luan.

**Writing – review & editing:** Yanli Li.
